# Cross-sectional imaging of aortic infections

**DOI:** 10.1007/s13244-016-0522-5

**Published:** 2016-10-19

**Authors:** D. J. Murphy, A. R. Keraliya, M. D. Agrawal, A Aghayev, M. L. Steigner

**Affiliations:** Division of Non-invasive Cardiovascular Imaging, Department of Radiology, Brigham and Women’s Hospital, 75 Francis Street, Boston, MA 02115 USA

**Keywords:** Computed tomography, Magnetic resonance angiography, Aorta, Endocarditis, Infection

## Abstract

Aortic infections are uncommon clinical entities, but are associated with high rates of morbidity and mortality. In this review, we focus on the cross-sectional imaging appearance of aortic infections, including aortic valve endocarditis, pyogenic aortitis, mycotic aneurysm and aortic graft infections, with an emphasis on CT, MRI and PET/CT appearance.

*Teaching Points*

• *Aortic infections are associated with high morbidity and mortality.*

• *CT, MRI and FDG PET/CT play complementary roles in aortic infection imaging.*

• *Radiologists should be vigilant for aortic infection manifestations to ensure timely diagnosis.*

## Introduction

Aortic infections are rare but significant clinical entities, associated with high rates of morbidity and mortality. It is crucial that reporting radiologists are alert to the various manifestations of aortic infections on cross-sectional imaging, as prompt diagnosis and treatment can be lifesaving. The imaging appearance of aortic infections varies based on the location of the infection, the culprit organism, the presence of aortic graft material and any other previous surgical intervention. In this review, we will describe the cross-sectional imaging appearance of the various types of aortic infections.

## Imaging modalities

Echocardiography, ultrasound, computed tomography (CT), magnetic resonance angiography (MRA) and fluorodeoxyglucose positron emission tomography (18 F-FDG PET/CT) are the principal noninvasive imaging modalities used to image the aorta.

Transthoracic echocardiography (TTE) is often the initial imaging modality in cases of suspected aortic valve infection. It is a quick, portable, non-invasive test with the benefit of a lack of ionizing radiation. Imaging of the aortic valve by TTE can be complicated by artefacts, especially in patients with valvular calcification or valve prostheses [1]. Transoesophageal echocardiography (TOE) is a more sensitive test for evaluating the aortic valve for suspected endocarditis, but has the disadvantage of being an invasive technique, often requiring sedation [2].

CT angiography (CTA) is a rapid, three-dimensional cross-sectional imaging technique offering excellent spatial and temporal resolution. The improved temporal resolution with modern scanners—as low as 66 ms in third-generation dual-source CT—allows for excellent anatomical assessment of the aortic valve [3]. The use of echocardiographic (ECG) gating when performing CT of the aortic valve or ascending thoracic aorta can help to improve image quality, minimizing the detrimental effect of cardiac motion, albeit at the cost of increased total radiation dose [4]. Although standard for aortic valve CT, ECG gating is not universally employed for imaging of the ascending thoracic aorta, especially when using modern dual-source scanners that offer improved temporal resolution. CT with arterial phase acquisition is our preferred initial cross-sectional imaging modality in cases of suspected aortic infection. In cases where there is high clinical suspicion of infection-related complications, such as pseudoaneurysm or fistula formation, we acquire an additional delayed phase (approximately 70 s).

MRA offers multi-parametric aortic imaging with excellent soft tissue contrast and tissue characterisation. Steady-state free precession (SSFP) cine imaging of the aortic valve provides both anatomical and functional information on the aortic valve. A temporal resolution of less than 50 ms is readily achievable, enabling accurate assessment of valve structure and function throughout the cardiac cycle [5]. Magnetic resonance imaging (MRI) is not our first-line cross-sectional modality for the assessment of suspected aortic valve infection, but it can be a useful tool in providing functional haemodynamic information which can inform treatment decisions. Contrast-enhanced MR angiography (CE-MRA) with gadolinium-based contrast agents provides excellent anatomical detail of the aorta with excellent soft tissue contrast, and without ionizing radiation [6]. Double inversion recovery (DIR) sequences null the blood pool, providing excellent visualisation of the aortic wall distinct from the aortic lumen [5]. Acquiring these images before and after administration of paramagnetic gadolinium-based contrast agents (GBCAs) enables an assessment of aortic wall enhancement, which can be an important feature in cases of aortic infection. We use MRA primarily in patients with equivocal findings on CT, in young patients, and for the serial follow-up of patients in order to reduce the radiation dose from CT.

The assessment of systemic infection/inflammation has emerged as a principal application of 18 F-FDG PET/CT outside oncology. Foci of active infection are often metabolically active, and will avidly take up glucose. The major role of 18 F-FDG PET/CT currently is as a problem-solving tool in equivocal cases, especially in cases of suspected aortic prosthetic infections [7].

Ultrasound provides a rapid assessment of aortic dimensions and information on the aortic wall, but outside of echocardiography it is not commonly used in cases of suspected aortic infection. Visualisation of the thoracic aorta beyond the aortic arch is often difficult on transthoracic ultrasonography due to an inability to image through bone and air, providing an incomplete anatomical assessment [8, 9]. TOE can overcome most of these limitations, although there is a short segment of the distal ascending aorta immediately proximal to the right brachiocephalic artery that remains obscured due to the interposition of the right bronchus and trachea creating an acoustic mismatch. Abdominal aorta sonography is operator-dependent, and can be limited by body habitus or overlying bowel gas [3, 10].

In this review, we will focus mainly on the role of CT, MRI and 18 F-FDG PET/CT in the assessment of aortic infections, with a brief discussion of the role of echocardiography and ultrasound.

## Aortic valve endocarditis

Infective endocarditis (IE) of the aortic valve is a serious, life-threatening condition, with a mortality rate of 30 % at 1 year [11]. It is caused by infection of the aortic valve, either native or prosthetic. The major risk factors for native valve endocarditis include degenerative valve disease, diabetes, cancer, intravenous drug use and congenital heart disease [12]. The epidemiology of IE has changed, with nosocomial IE now accounting for approximately 30 % of cases, largely due to the increased use of long-term intravenous lines, invasive procedures, prosthetic valves and indwelling cardiac devices [1, 13]. Gram-positive cocci (*Staphylococcus*, *Streptococcus* and *Enterococcus*) account for 80–90 % of cases, with *S. aureus* the most frequently isolated organism, causing up to 30 % of cases (Table 1) [12, 13].Staphylococcal IE is no longer limited to the traditional at-risk groups such as patients with renal failure on haemodialysis or intravenous drug users, and it can affect both native and prosthetic valves [14].

**Table 1:** Common causative organisms in aortic infections

Diagnosis	Organism
Native aortic valve endocarditis [12–14]	*Staphylococcus aureus* Viridans streptococci (*S. mutans*, *S. salivarius*, *S. mitis*) Coagulase-negative staphylococci (*S. epidermidis*, *S. lugdunensis*, *S. capitis*) Group D streptococci (*S. bovis*)
Prosthetic aortic valve endocarditis [12–14]	Coagulase-negative staphylococci *S. aureus* *Enterococcus* sp. (*E. faecalis*) Fungi (*Candida* or *Aspergillus*)
Infectious aortitis [47, 49–51]	*Salmonella* sp. *S. aureus* *S. pneumoniae* *E. faecalis* *M. tuberculosis* Viral (HIV) Fungi (*Candida* or *Aspergillus*) *T. pallidum*
Aortic graft infection [82–84]	Early (0–90 days): *S. aureus* Late (>90 days): Coagulase-negative staphylococci

IE has myriad of clinical presentations, including pyrexia of unknown origin, stroke and systemic emboli. Definitive diagnosis requires integration of clinical, laboratory and imaging results, incorporated into the modified Duke criteria [15, 16]. Echocardiography is often the initial imaging modality utilized in cases of suspected IE. The major features of endomyocardial valvular infection on echocardiography include the presence of valvular vegetations, periannular tissue destruction, abscess, aneurysms, fistulas, leaflet perforation or valvular dehiscence [17]. Transthoracic echocardiography (TTE) is a moderately sensitive and highly specific test (75 % and 90 %, respectively) for the presence of vegetations in suspected native valve endocarditis [1, 17]. TOE is the current imaging gold standard, with sensitivity of more than 90 %, and is useful in cases with a moderate to high clinical suspicion and negative TTE [1, 18]. Both TTE and TOE can be limited by factors such as patient habitus, variant anatomy, artefacts from heavy valve calcification and the presence of metal prosthetic valves [17, 19].

CT provides aortic valve imaging with high spatial and temporal resolution. It is an excellent imaging option in patients with suspected IE, particularly in those with a negative TTE who are too high risk to undergo a TOE. A series by Feuchtner et al. compared the diagnostic performance of ECG-gated CT and TOE in patients with clinically suspected IE, with CT performing comparatively well, with sensitivity of 97 % and specificity of 88 % [20].

On CT vegetations appear as irregularly shaped, low-attenuation masses adherent to the valve or endomyocardial surface, which usually oscillate throughout the cardiac cycle [21, 22]. Vegetation size and mobility are the most important factors for determining the risk of cerebrovascular or systemic embolism. The vegetation size is defined by its maximal length, with those >10 mm carrying a high risk of embolism [17]. CT has high sensitivity in detecting these large vegetations, with one series reporting 100 % sensitivity for lesions larger than 10 mm [22]. The detection of vegetations by CT is challenging when there are existing calcified degenerative lesions and when the vegetations are small, especially when <2 mm in size [18]. Large vegetations may cause perforation of the aortic valve leaflets, which is readily visible on CT (Table 2). The 2015 European Society of Cardiology (ESC) guidelines on the diagnosis and treatment of IE have proposed the presence of a paravalvular lesion on CT as a major criterion in the modified Duke criteria [17].

**Table 2:** Aortic infections: CT, MR and 18 F FDG-PET/CT imaging features, advantages and disadvantages

Infection	Key imaging features	CT *Advantages*	*Limitations*	MRI *Advantages*	*Limitations*	FDG-PET/CT *Advantages*	*Limitations*
AV endocarditis [20–22, 25, 26]	• Leaflet vegetation • Leaflet perforation • Perivalvular abscess • IFB extension • Pseudoaneurysm	• Vegetations >2 mm oscillating & complications	• Increased radiation dose with multiphase CT	• Functional assessment	• Lower spatial resolution than CT • Flow-related artefacts	• Distal septic emboli	• Low sensitivity (66 %)
PV endocarditis [36, 44–46]	• Valve dehiscence • Perivalvular abscess • Pseudoaneurysm	• Valve dehiscence on multiphase CT	• Increased radiation dose with multiphase CT • Prosthesis-related artefacts	• Functional assessment	• Prosthesis-related artefacts • Flow-related artefacts	• Increases specificity of modified Duke criteria to 91–97 % • Distal septic emboli	• Not evaluable until >90 days post-insertion
Infectious aortitis [47, 49, 57]	• Aortic wall thickening (often crescentic) ± enhancement • Pockets of gas • Adjacent fat stranding & fluid	• Demonstrates gas & periaortic inflammation	• Aortic mural enhancement can be hard to appreciate	• Superior aortic wall assessment • Sensitive for periaortic abscess	• Gas & surrounding inflammatory changes harder to define	• Small series reports sensitivity of 100 %	• Low spatial resolution
Mycotic aneurysm [47, 49, 54, 59]	• Saccular aneurysm • Rapid growth in aneurysm size • Adjacent fat stranding & fluid	• Periaortic inflammation easily demonstrated	• Increased radiation if multiple sequential studies	• Lack of radiation in sequential studies	• Peri-aneurysm inflammation less well defined than on CT	• Can distinguish infected from bland aneurysm	• Low spatial resolution
Aortic graft infection [44, 45, 49, 92, 94–96]	• Pseudoaneurysm • Perigraft soft tissue stranding • Perigraft fluid & gas • Aortoenteric fistula	• Periaortic inflammation is clearly visible • Can demonstrate fistula	• Increased radiation dose with multiphasic CT	• Can distinguish perigraft fluid from haematoma	• Prosthesis-related artefacts	• Reported sensitivity of 89–100 %	• False positives in early postoperative phase

The role of cardiac MRI (CMR) in the initial diagnosis of suspected aortic valve endocarditis is limited. Vegetations are best depicted on SSFP cine imaging as low signal masses attached to the valve surface or endocardium, which oscillate during the cardiac cycle. The lower spatial resolution of CMR compared to CT or TOE limits its diagnostic role in suspected IE. The presence of an off-resonance artefact, particularly at 3.0 Tesla, can hinder the use of SSFP sequences in aortic valve imaging, often requiring the use of gradient echo (GRE) cine imaging [23]. There is little data on the performance of CMR in detecting vegetations, but it can provide haemodynamic information. CMR can help quantify the severity of aortic insufficiency, if present, which can be useful in triaging patients with endocarditis to medical or surgical treatment (Table 2) [17]. A brain MRI with GBCA is useful in patients with suspected IE and neurological symptoms, providing superior detection and characterisation of lesions compared to CT, with reported sensitivity for clinically symptomatic cerebral lesions of 100 %, versus 81 % for contrast-enhanced CT [24].

18 F-FDG PET/CT can be used to visualise vegetations, which will avidly take up the glucose tracer. A recent meta-analysis on the use of 18 F-FDG PET/CT for native valve IE (at any valve) reported a pooled sensitivity and specificity of 61 % and 88 %, respectively [25]. Although this sensitivity is not comparable to echocardiography or CT for native aortic valve endocarditis, 18 F-FDG PET/CT can be used as a problem-solving tool in cases with high clinical suspicion and negative imaging, and in the detection of distant septic emboli (Table 2) [26–28].

The major differential diagnoses of aortic valve vegetations are thrombi, papillary fibroelastoma, myxomatous changes and giant Lambl’s excrescences [29]. Papillary fibroelastomas are usually attached to the aortic side of the valve and can potentially cause coronary ostial obstruction, whilst vegetations are often attached to the free end of the valve [30]. These tumours typically enhance on CMR following gadolinium administration, which can be another distinguishing feature [31]. Giant Lambl’s excrescences are formed by the coalescence of multiple filiform fronds, which form at sites of valve closure likely resulting from endothelial wear and tear [32]. They are associated with thrombi, and can cause embolic events [33, 34]. Overall, there is significant overlap in imaging features between these entities and vegetations. Incorporating the imaging findings with clinical and laboratory data is thus crucial in accurately diagnosing IE.

### Local endocarditis-related complications

Extension of infection into the perivalvular tissue can manifest as abscesses, pseudoaneurysms and fistulas. These complications occur in 10–40 % of cases of native aortic valve IE, necessitating surgical treatment [35]. CT is excellent in detecting perivalvular extension, with one series demonstrating 100 % sensitivity, using surgery as a reference standard [20]. Perivalvular abscess appears on CT as a collection of fluid density around the aortic valve surrounded by inflammatory tissue, which can enhance. An abscess can spread into surrounding structures, such as the interatrial septum or left ventricular myocardium. This can manifest clinically as atrioventricular (AV) nodal block, new bundle branch block or persistent sepsis. Rupture of a perivalvular abscess into the aortic root creates a pseudoaneurysm. This manifests on CT as an abnormal contrast-containing cavity adjacent to the aortic valve, which freely communicates with the aortic lumen.

**Fig. 1:**
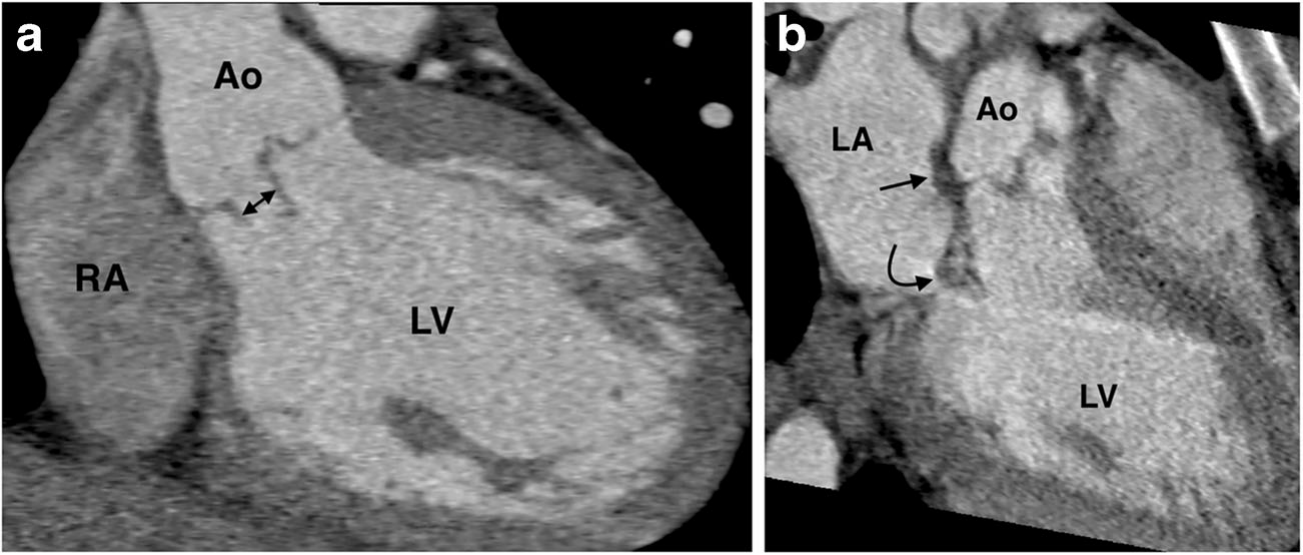
An ECG-gated cardiac CT angiogram in a 22-year-old female intravenous drug user with a bicuspid aortic valve, fever and a new murmur. (**a**) A coronal multiplanar reformat (MPR) of the aortic valve demonstrates a perforation (*double arrow*) in the posterior leaflet of the bicuspid aortic valve. (**b**) A 3-chamber MPR demonstrates periaortic soft tissue thickening posterior to the aortic root (*arrow*), extending inferiorly towards the anterior leaflet of the mitral valve (*curved arrow*), consistent with spread of infection to the intervalvular fibrous body. The patient underwent aortic and mitral valve replacement, and the infected valve tissue grew *S. aureus. LA = left atrium; LV = left ventricle; Ao = ascending aorta; RA = right atrium; RV = right ventricle*

Extension of a pseudoaneurysm or abscess into the intervalvular fibrous body (IFB) is an important finding which may have implications in surgical planning, and one which can be readily assessed on CT (Fig. 1) [22, 36, 37]. The IFB is a fibrous structure located between the lateral and medial fibrous trigones, connecting the anterior mitral valve leaflet and the left and non-coronary aortic valve cusps to the heart’s fibrous skeleton. Destruction of the IFB by an aortic root abscess or pseudoaneurysm complicates surgical intervention, removing the inherent local stability required to successfully perform an aortic valve replacement. In these cases, radical debridement of all the infected tissue with reconstruction of the IFB, followed by aortic and mitral valve replacement, is the only surgical option [38]. Fistula formation is a rare and serious complication of aortic valve IE, occurring when a perivalvular abscess or pseudoaneurysm ruptures into an adjacent cardiac cavity.

### Choosing an imaging modality

**Fig. 2:**
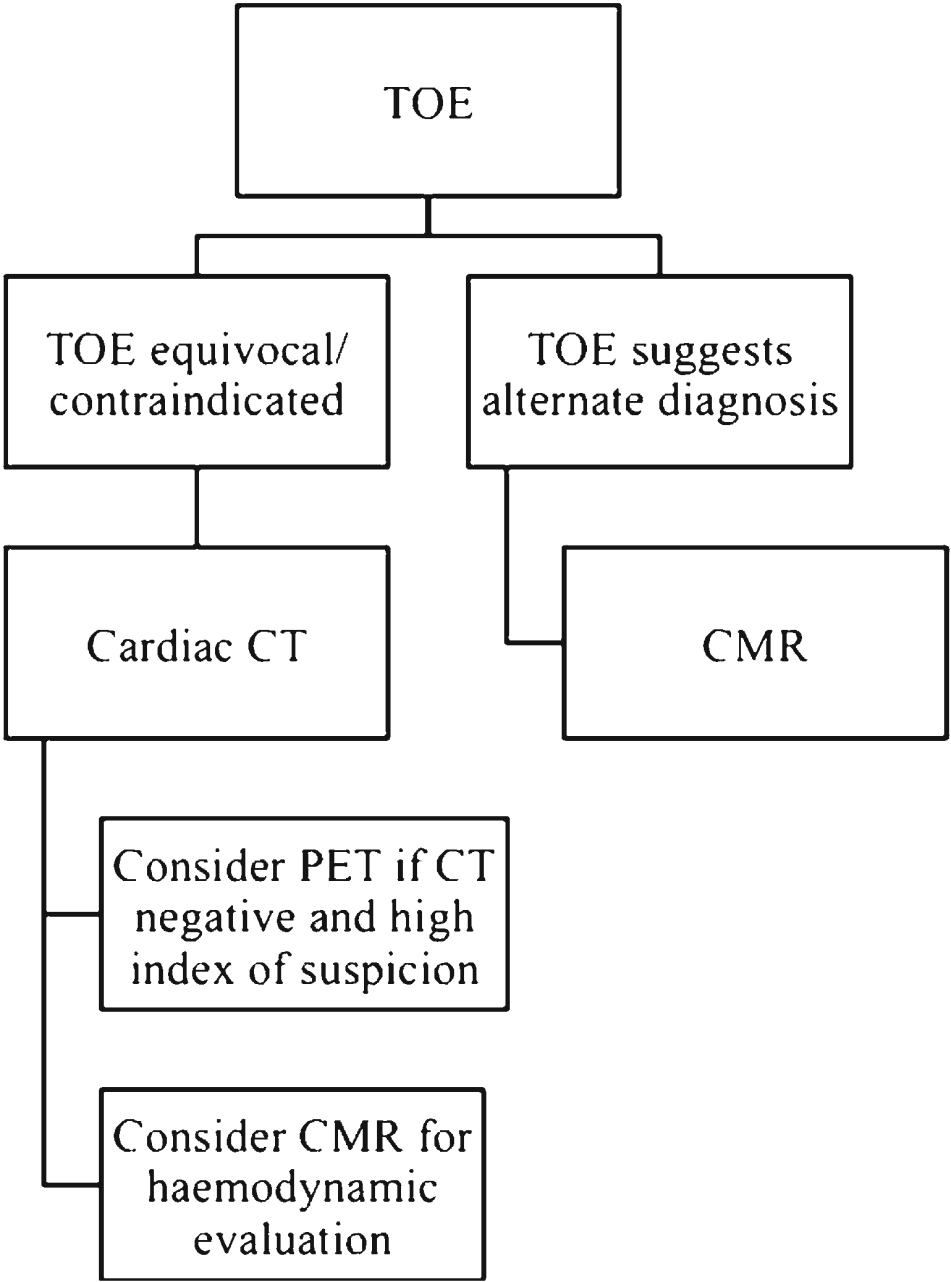
A suggested imaging workflow diagram for suspected native aortic valve endocarditis with a negative TTE [17]

TTE and TOE remain the initial imaging modalities of choice for evaluating cases of suspected aortic valve IE, with CT the favoured next line of imaging (Fig. 2) [17]. CT is currently reserved mainly for patients in whom diagnosis is equivocal on TTE and who cannot undergo a TOE, or in cases of suspected local IE-related complications that may require surgical intervention, as outlined above [17, 20]. For these cases, we perform an ECG-gated cardiac CT angiogram (arterial phase acquisition with approximately 60 mL iodinated contrast), with acquisition throughout the entire R-R wave interval; this allows assessment of the aortic valve and adjacent structures throughout the cardiac cycle, and can demonstrate oscillation of leaflet vegetations if present. Acquiring CT data throughout the entire R-R wave interval is associated with increased radiation dose compared with ECG-gated CT acquisitions limited to either end-systole or end-diastole [39]. To help mitigate this effect, a number of dose-saving steps can be taken. These include the use of ECG-based tube current modulation, scanning at reduced kilovoltage (kVp), such as 80/100 kVp rather than 120 kVp, reducing scan z-axis coverage, and the use of advanced image reconstruction techniques such as iterative reconstruction [4, 40, 41]. The role of CMR in the primary diagnosis of IE is limited, but it is a useful imaging choice when different aortic valve pathology such as tumour or thrombus is suspected. We typically reserve the use of 18 F-FDG PET/CT for patients in whom there is high clinical suspicion but previous negative imaging findings.

### Prosthetic valve endocarditis

Aortic valve prosthetic valve endocarditis (PVE) has a poor prognosis, with a reported mortality of 20–40 % [42]. This is likely due to the higher propensity for infection to extend into the perivalvular tissue compared with native valve endocarditis [2, 17]. Bioprosthetic valves (also referred to as tissue valves) are predominantly composed of soft tissue material (usually porcine xenograft), and when infected have an imaging appearance similar to that described in native valve infection.

**Fig. 3:**
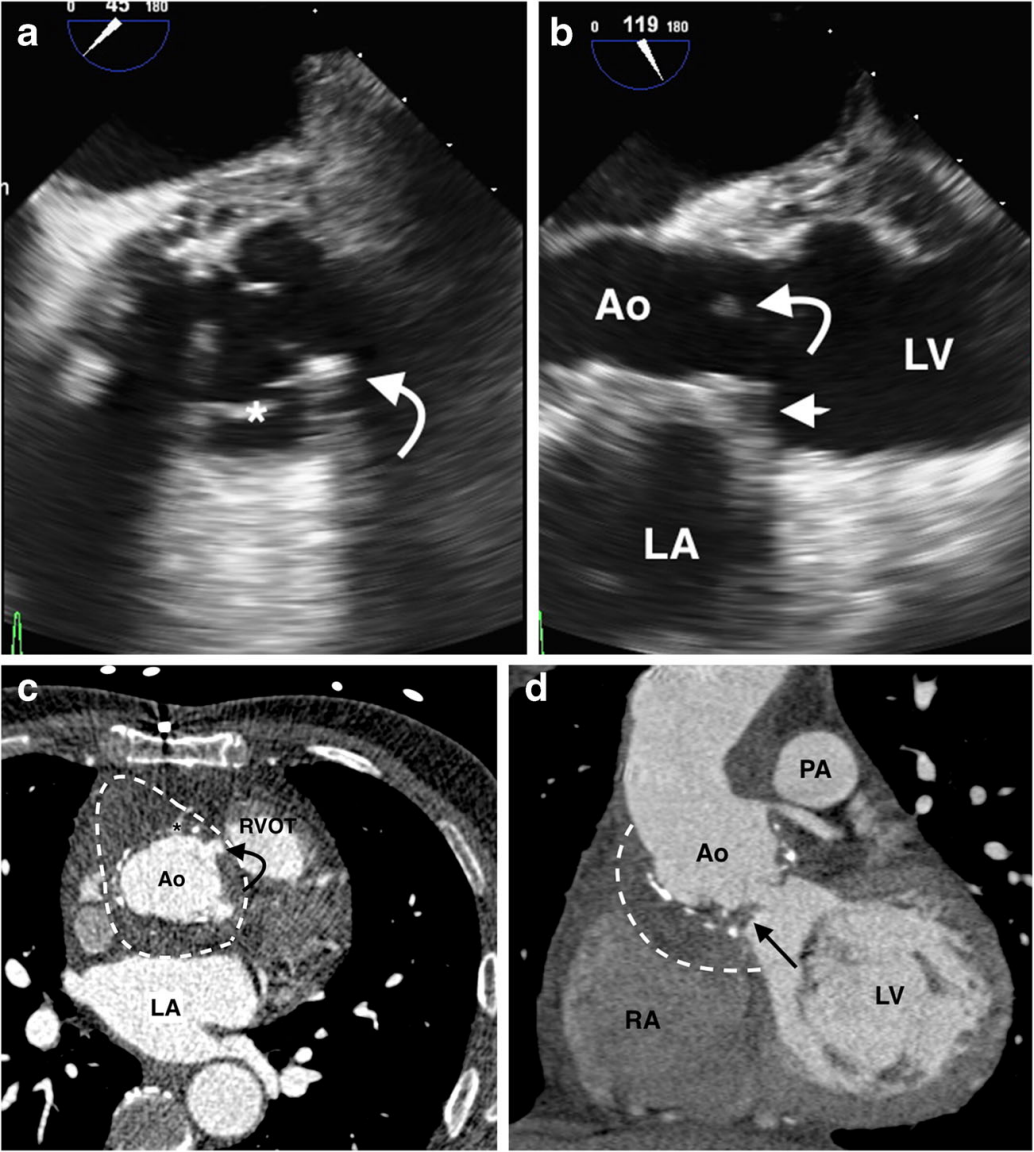
A 65-year-old man with a previous history of bioprosthetic aortic valve replacement and ascending aorta homograft repair presents with fever and sepsis. (**a**) A still TOE image short axis to the aortic valve demonstrates an echodensity (*curved arrow*) arising from the non-coronary cusp (*) of the prosthetic aortic valve; this echodensity demonstrated mobility on real-time cine imaging consistent with a vegetation. (**b**) A 3-chamber TOE still image re-demonstrates the prosthetic aortic valve vegetation (*curved arrow*), with echogenic soft tissue thickening of the posterior aortic valve annulus (*straight arrow*), concerning for paravalvular abscess. (**c**) An axial ECG-gated cardiac CT angiogram demonstrates a large aortic root abscess (*dashed line*) with a small pseudoaneurysm (*curved arrow*) arising from the right sinus of Valsalva, adjacent to the origin of the right coronary artery (*). (**d**) A coronal MPR from the CT re-demonstrates the vegetation on the non-coronary cusp of the bioprosthetic valve (*arrow*), with further delineation of the aortic root abscess (*dashed line*). The patient underwent a repeat aortic valve and ascending aorta homograft replacement. Tissue culture of the removed bioprosthetic valve grew coagulase-negative *Staphylococcus* and *E. faecalis*. *Ao = ascending aorta; LA = left atrium; RA = right atrium; PA = pulmonary artery; RVOT = right ventricular outflow tract*

**Fig. 4:**
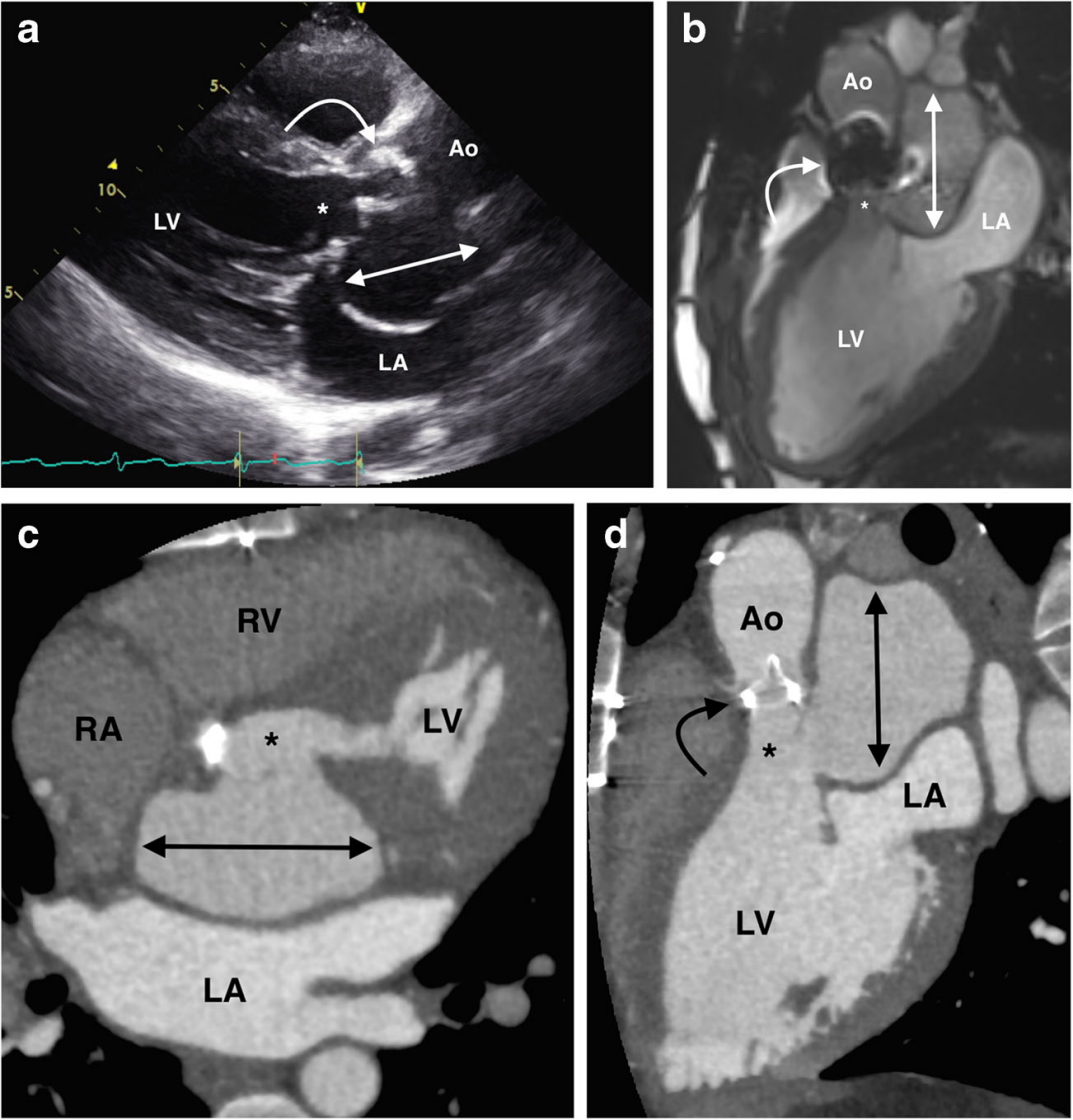
A 34-year-old man presents with dyspnoea; he has a history of intravenous drug use, having previously undergone an aortic valve replacement for *S. aureus* endocarditis. (**a**) A still 3-chamber image from a TTE demonstrates a large hypoechoic fluid collection (*double arrows*) between the prosthetic aortic valve (*curved arrow*) anteriorly and the left atrium posteriorly, with possible communication with the left ventricular outflow tract [LVOT (*)]. (**b**) A 3-chamber CMR SSFP image demonstrates a large pseudoaneurysm (*double arrows*) arising from the LVOT (*), a sequela of prosthetic valve endocarditis. Note the artefact from the aortic valve prosthesis (*curved arrow*). An (**c**) axial ECG-gated cardiac CT angiogram and (**d**) 3-chamber CT MPR demonstrates the large pseudoaneurysm (*double arrows*) arising from the LVOT (*), inferior to the prosthetic aortic valve (curved arrow). The patient underwent surgical aortic root replacement and pseudoaneurysm repair. *Ao = aorta; LA = left atrium; LV = left ventricle; RA = right atrium; RV = right ventricle*

Mechanical prosthetic valve imaging by echocardiography can be limited by artefacts caused by acoustic shadowing. IE is suspected on a prosthetic valve when there is a perivalvular mass or when valve dehiscence can be demonstrated. ECG-gated CT performs well in the identification of PVE, with one series reporting sensitivity of 93 % when compared to surgical findings [36]. The major signs of PVE on CT are the presence of vegetations, thickening of the aortic root wall of >5 mm, the presence of a perivalvular abscess or pseudoaneurysm, and prosthetic valve dehiscence (Figs. 3, 4). The latter manifests as a rocking motion of the mechanical valve throughout the cardiac cycle, which can be demonstrated on ECG-gated cardiac CT with multiphase acquisition throughout the cardiac cycle [21, 36]. Transcatheter aortic valve replacements (TAVR) are a novel category of mechanical aortic valves, often placed in patients who are deemed too high-risk to undergo open aortic valve replacement. Cases of post-TAVR endocarditis have an appearance on CT similar to that with mechanical aortic PVE [43].

**Fig. 5:**
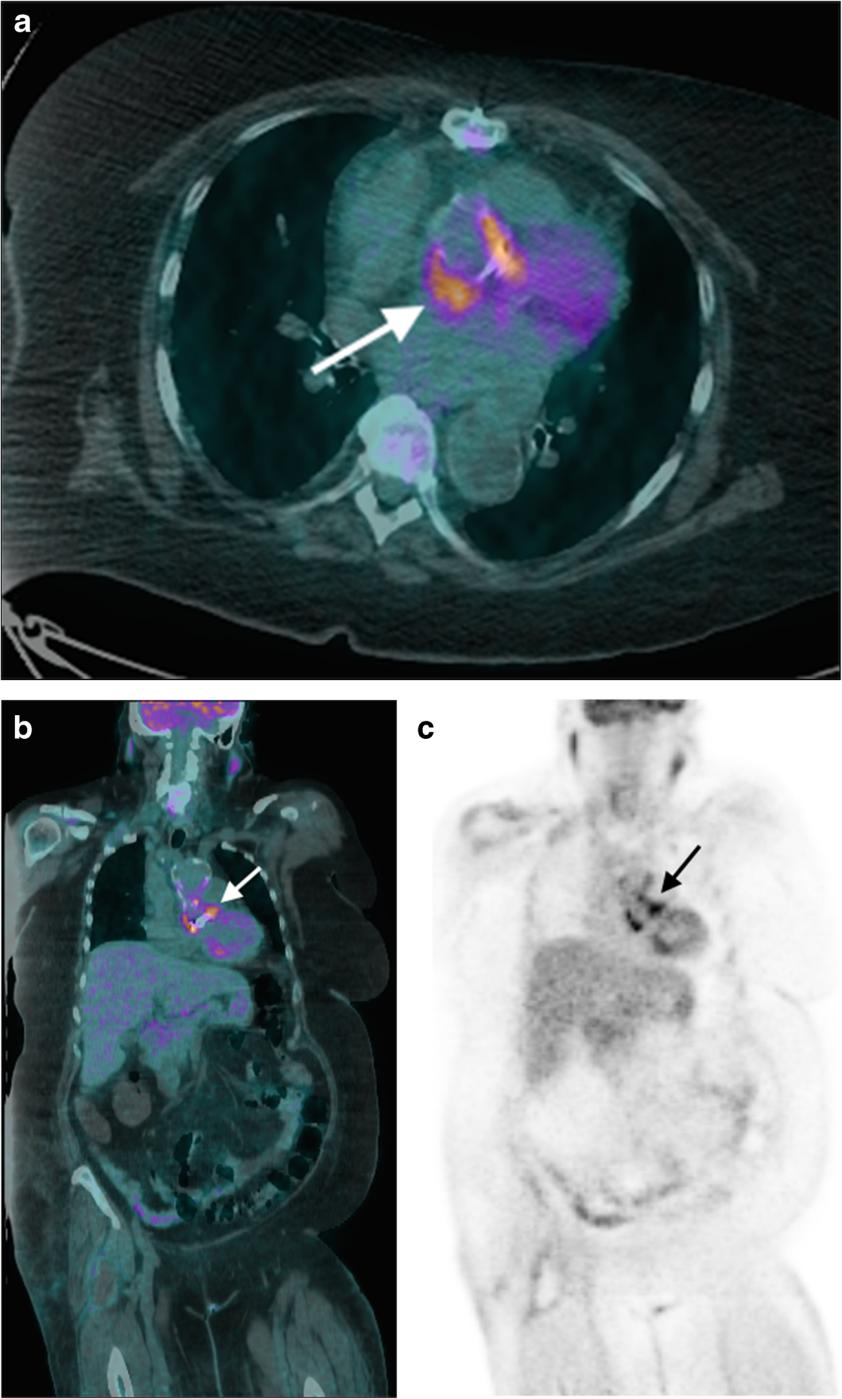
A 65-year-old woman presents with fever and dyspnoea 6 years post-mechanical aortic valve replacement and aortic root replacement for a type A aortic dissection. Blood cultures grew *S. aureus*, and the patient was referred for a TOE, which was equivocal. (**a**) Axial and (**b**) coronal fused 18 F-FDG PET/CT images and (**c**) coronal unfused 18 F-FDG PET images demonstrate intense FDG around the prosthetic aortic valve and at the aortic root (*arrow*) consistent with prosthetic valve endocarditis. The patient underwent reoperation for aortic valve replacement and aortic root repair

A systematic review of the role of non-invasive imaging in the diagnosis of PVE supports the use of CT in addition to echocardiography for improving diagnostic accuracy, especially in cases of life-threatening perivalvular extension [19]. One of the main limitations of CT in the assessment of PVE is the presence of a streak artefact caused by the high-density valve material. Artefacts caused by valve material often limit the role of CMR in the diagnosis of mechanical PVE, causing local spin dephasing (Table 2). Given these limitations, 18 F-FDG PET/CT has emerged as a useful adjunct in the diagnosis of PVE (Fig. 5). Studies by Saby et al. and Pizzi et al. demonstrated that the addition of increased tracer uptake around the prosthetic valve on 18 F-FDG PET/CT in cases of suspected PVE increased the sensitivity of the modified Duke criteria from 70 to 97 % and 52 to 91 %, respectively [44, 45]. A recent study by Fagman et al. suggests that a semiquantitative PET/CT analysis may be useful in cases of suspected PVE; the authors calculated a ratio of maximal standard uptake values (SUV_max_) in the valve area and descending aorta, demonstrating a significant association between a valve/descending aorta SUV_ratio_ >1.7 and infection [46]. The current 2015 ESC IE guidelines recommend that the presence of abnormal increased activity on a prosthetic valve by 18 F-FDG PET/CT should be considered a major criterion for PVE diagnosis, once the valve has been implanted for more than 3 months [17].

### Choosing an imaging modality

**Fig. 6:**
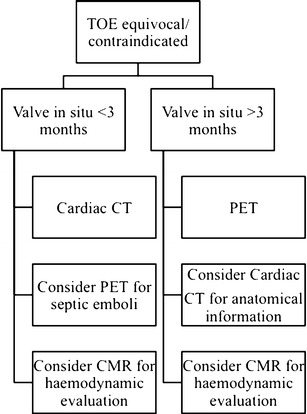
A suggested imaging workflow diagram for suspected prosthetic aortic valve endocarditis with a negative TTE [17]

TOE is the imaging modality of choice in cases of suspected PVE, although its diagnostic value can be limited by artefacts, and a negative echocardiogram does not exclude the diagnosis [17]. Additional imaging is necessary both in patients with a negative TOE and high clinical suspicion, such as those with new periprosthetic regurgitation, as well as in patients that cannot undergo TOE. ECG-gated cardiac CT angiography (with full R-R wave interval acquisition) and 18 F-FDG PET/CT are the next imaging modalities of choice, with the latter reserved primarily for patients with a prosthesis that has been implanted for longer than 3 months (Fig. 6) [17].

## Infectious aortitis

The aortic intima is normally very resistant to infection, with most cases of aortic wall infection occurring in patients with an underlying mural abnormality, such as atherosclerosis, cystic medical necrosis or in the presence of an aortic prosthesis [47–49]. Bacteria are the most common culprit microorganisms, especially *Salmonella*, *Enterococcus* and *Staphylococcal* species, and are often associated with a concurrent episode of gastroenteritis or osteomyelitis [10, 50, 51]. Other causative organisms include *Streptococcus pneumoniae*, *Listeria*, *Bacteroides fragilis*, *Clostridium*, human immunodeficiency virus (HIV), *Mycobacterium tuberculosis* and *Treponema pallidum* (Table 1) [47, 49]. Fungal aortic infection is uncommon, usually occurring in immunosuppressed patients, such as those on chemotherapy or HIV patients.

### Pyogenic aortitis

Bacterial aortic wall infection usually occurs when a segment of the wall is seeded by bacteria via the vasa vasorum [51]. This can result from systemic septic embolism, haematogenous seeding, spread from an adjacent focus of infection or by iatrogenic direct inoculation. The clinical presentation is determined by the site and extent of infection, varying from back or abdominal pain with fever to acute severe acute aortic regurgitation. *S. pneumoniae* and *Enterococcus* are commonly implicated in thoracic aortic infection, whilst *Salmonella* is the most common cause of infectious abdominal aortitis [52]. Prompt diagnosis is crucial, with mortality rates of up to 44 % despite treatment [53].

**Fig. 7:**
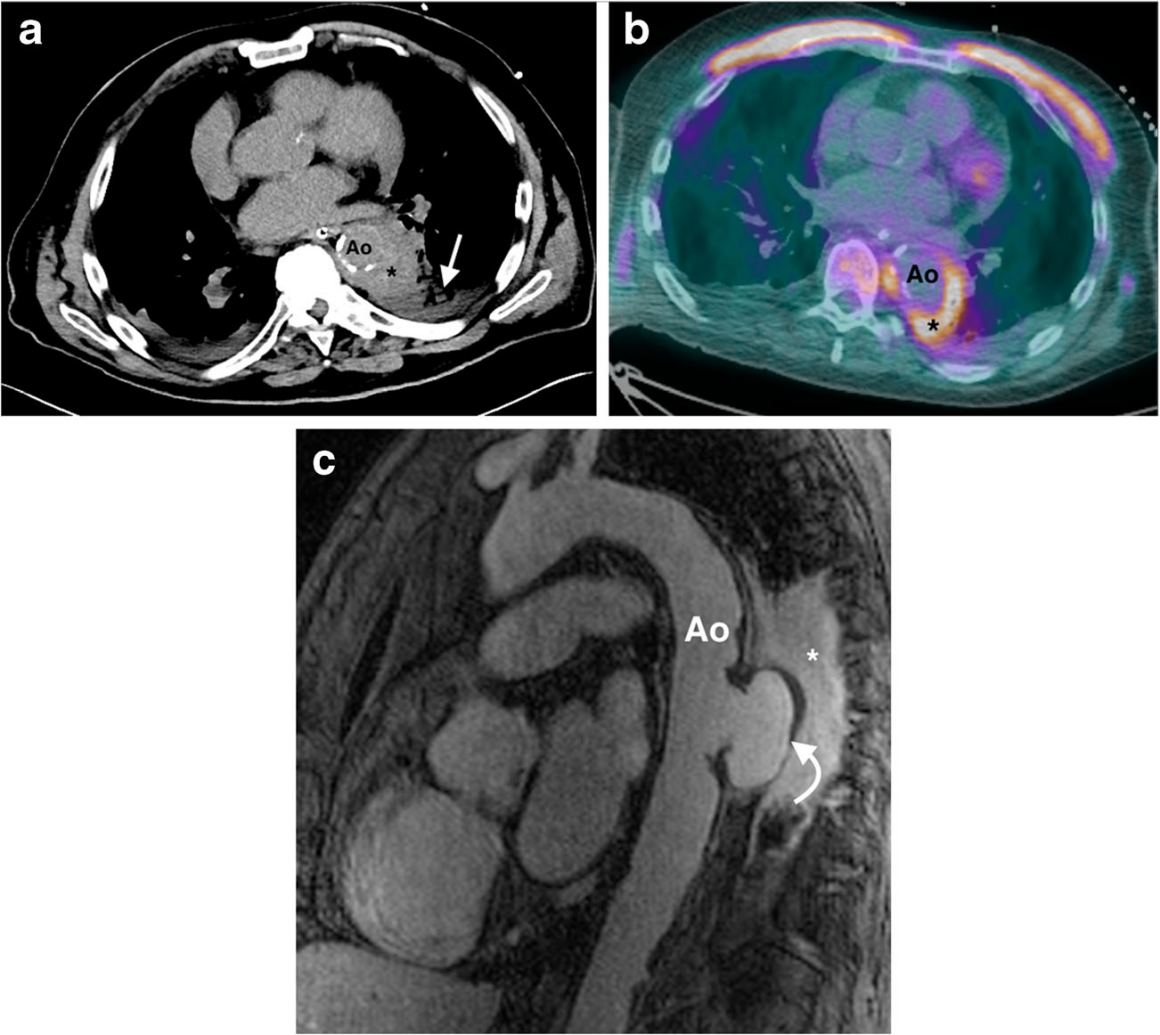
A 78-year-old man presents with chest pain, fever and *S. aureus* septicemia. (**a**) An axial non-contrast CT thorax image demonstrates high attenuation in the descending thoracic aorta wall (*), with adjacent consolidation in the left lower lobe (*arrow*). (**b**) An 18 F-FDG PET/CT image shows corresponding increased FDG uptake in the aortic wall (*). The patient was diagnosed with an infected intramural haematoma, and was treated with antibiotics. (**c**) A follow-up sagittal fat-saturated T1 MRI post-GBCA image shows development of a large saccular pseudoaneurysm (*curved arrow*), with persistent adjacent inflammatory tissue (*). The patient subsequently underwent surgical repair. *Ao = descending thoracic aorta*

**Fig. 8:**
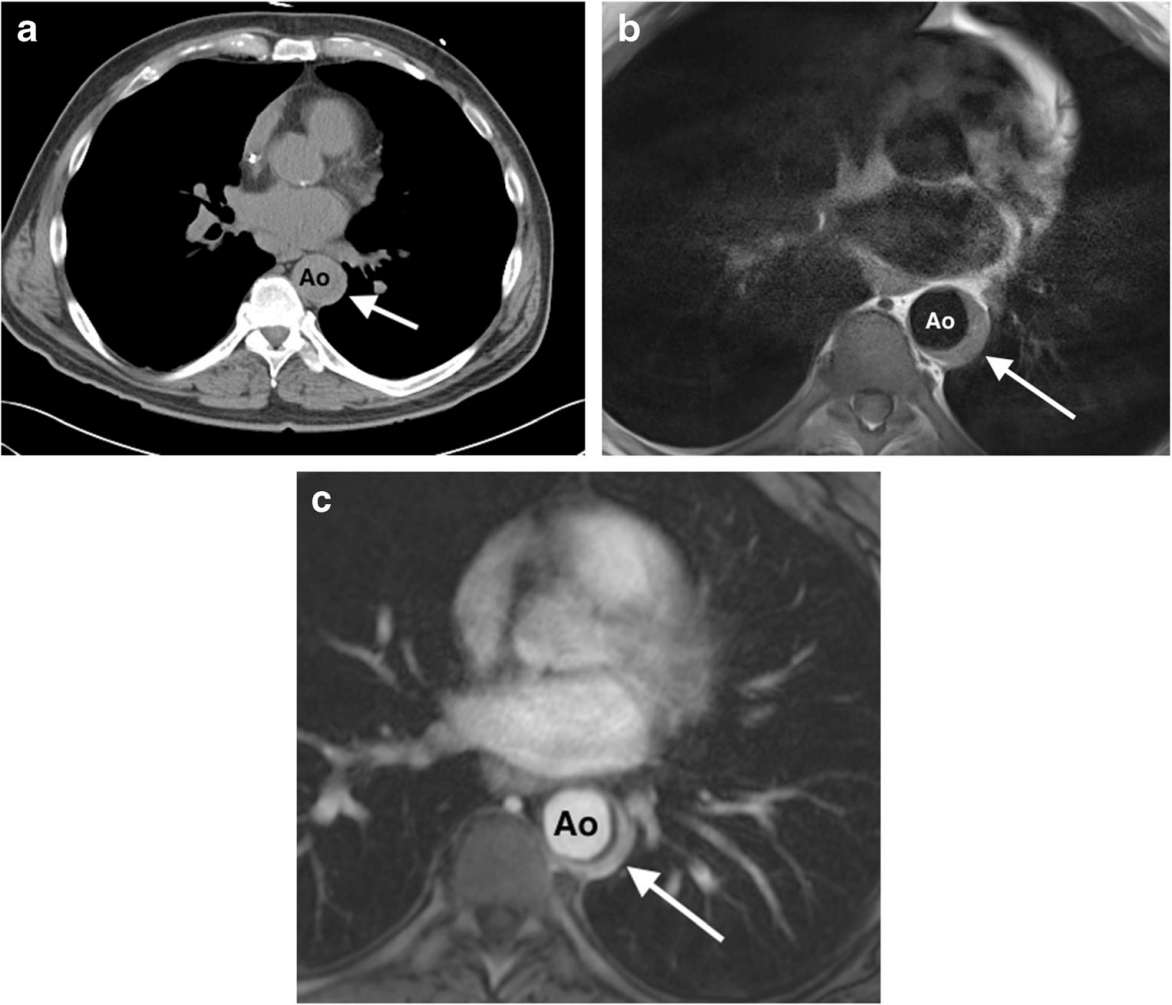
A 55-year-old man with a history of chronic myeloid leukemia presents with fever and chest pain. (**a**) An axial non-contrast CT scan of the thoracic aorta demonstrates crescentic mural thickening (*arrow*) of the mid-descending thoracic aorta. (**b**) An axial T1 double inversion recovery (DIR) MRI pre-contrast image demonstrates crescentic mural high signal in the same location in the descending thoracic aorta. (**c**) An axial T1 MRI post-GBCA image demonstrates crescentic mural enhancement in the corresponding location, consistent with an aortitis. The patient had new cryptococcal sepsis, with no other focus of infection, and a diagnosis of cryptococcal aortitis was made. The patient died despite systemic antifungal treatment. *Ao = descending thoracic aorta*

**Fig. 9:**
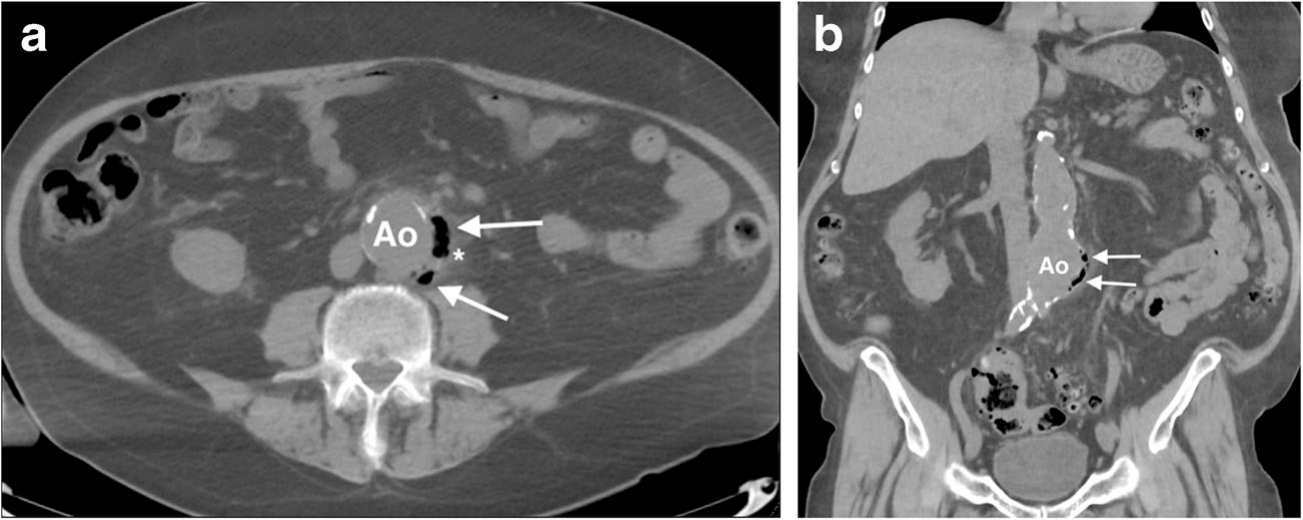
A 75-year-old man with a history of chronic kidney disease and type 2 diabetes mellitus presents with back pain and fever. (**a**) Axial and (**b**) coronal non-contrast CT scans of the abdomen demonstrates aneurysmal dilatation of the infrarenal aorta, with multiple pockets of gas within the aortic wall (*arrow*) and periaortic fat stranding (*), consistent with emphysematous aortitis. The patient underwent urgent excision of the mycotic aneurysm, with placement of an axillobifemoral bypass graft. The resected aortic wall grew *Clostridium perfringens*, a gram-positive, gas-forming anaerobic bacillus. *Ao = abdominal aorta*

CT or MR aortography are the initial imaging modalities of choice in many centres. CT is more commonly utilized due to greater availability and the shorter acquisition time in patients who are commonly systemically unwell. Signs of pyogenic aortitis on cross-sectional imaging include aortic wall-thickening (often asymmetric or crescentic rather than circumferential) and enhancement, periaortic fluid, periaortic soft tissue, rapidly enlarging saccular-type aortic aneurysm/pseudoaneurysm and air in the aortic wall (Figs. 7, 8) [47, 49, 54]. Periaortic oedema on CT manifests as either fat stranding or a low-attenuation rim surrounding the aorta, and on MRI as periaortic T2 hyperintensity [9, 10]. Periaortic inflammatory soft tissue appears as a homogeneously high attenuation mass on post-contrast CT, and on MRI is a high T2 signal and enhances homogeneously on T1 post-GBCA [10, 47]. Aortic mural gas is easier to appreciate on CT than MRI (Fig. 9), whilst aortic wall enhancement is often better visualized on MRI, especially using a T1-weighted DIR sequence pre- and post-GBCA administration. 18 F-FDG PET/CT can be used to detect inflammation and infection along the aorta. Elevated tracer uptake along the aortic wall is associated with aortic wall inflammation in large vessel vasculitis [55, 56], and a similar appearance has been described in pyogenic aortitis [57]. Aortitis on ultrasound can manifest as mural thickening with a hypoechoic periaortic mass, but there is limited data on the specific sonographic appearance in pyogenic aortitis [9, 10, 47, 49, 54]. The potential complications of pyogenic aortitis include septic emboli, mycotic aneurysm formation, aortic rupture and fistula formation.

### Mycotic aneurysms

**Fig. 10:**
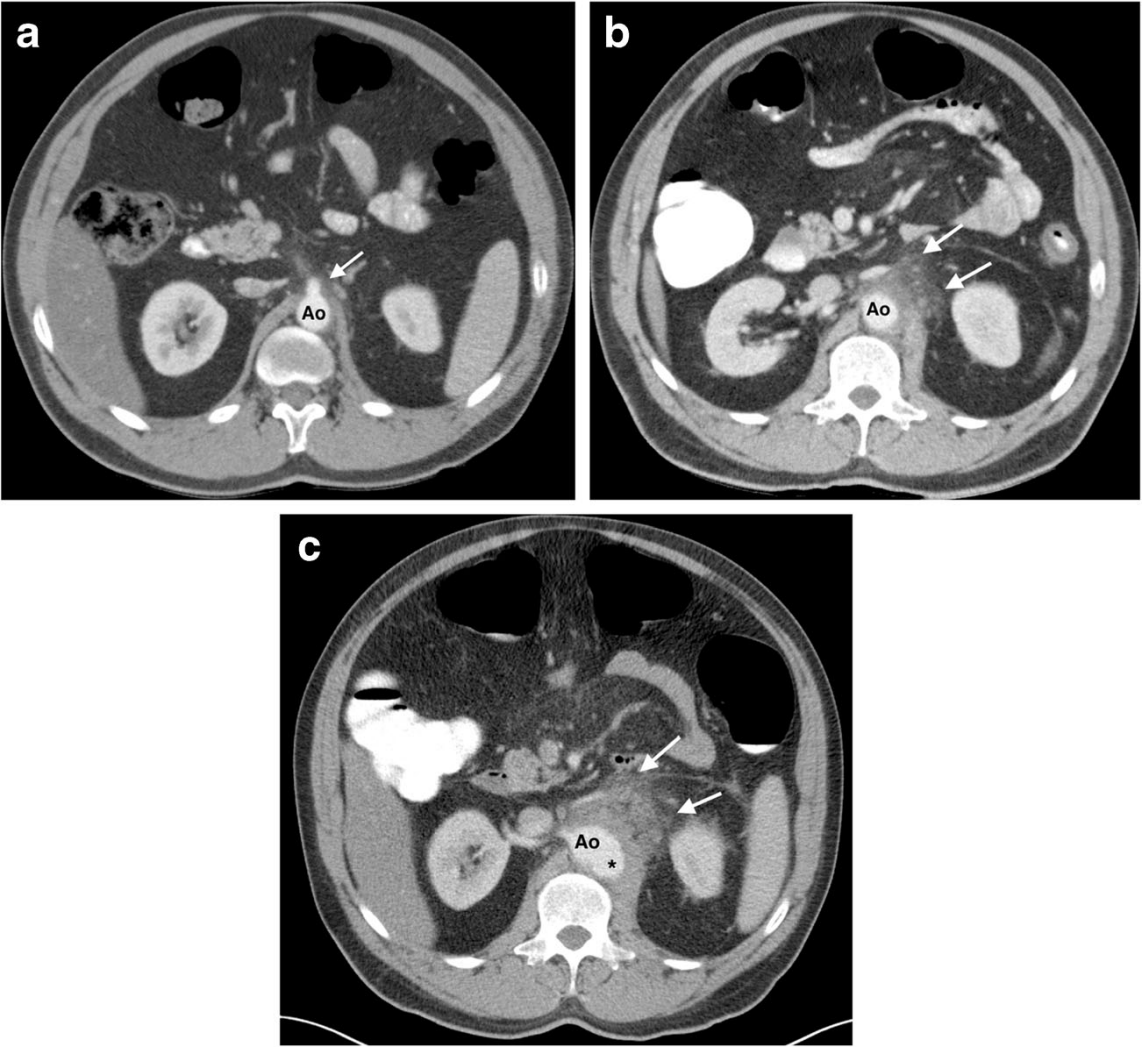
A 46-year-old man with no significant medical history presents with abdominal pain and low-grade fever. (**a**) An axial CT abdomen post-contrast image demonstrates ill-defined fat stranding (*arrow*) surrounding the proximal abdominal aorta at the level of the celiac artery origin. (**b**) A follow-up axial post-contrast CT scan of the abdomen performed 3 days later for worsening abdominal pain shows an interval increase in the severity of periaortic fat stranding (*arrows*). (**c**) A further follow-up axial post-contrast CT scan of the abdomen 1 week later shows the development of a saccular aortic aneurysm (*) with worsening periaortic fat stranding (*arrows*). The patient underwent surgical excision of the mycotic aneurysm with bypass grafting, and the excised aortic wall grew *S. aureus*. Despite surgical intervention, the patient died in the early postoperative period. *Ao = abdominal aorta*

The term ‘mycotic aneurysm’ can be a source of confusion., as it refers to all aortic aneurysms caused by infection, regardless of the culprit microorganism. These are rare entities, constituting only 0.7–2.6 % of all aortic aneurysms [54]. They usually result from an infectious aortitis, which weakens the wall, causing a contained aortic rupture and formation of a pseudoaneurysm. They most commonly affect the infrarenal abdominal aorta, with *Salmonella* the most common causative organism [54, 58]. They typically appear on CT and MRI as saccular aortic aneurysms with lobulated contours and periaortic soft tissue stranding and oedema/fluid, and may enlarge rapidly over a short period of time (often weeks) (Fig. 10) [10, 47, 49, 54]. Asymmetric periaortic fat density or a periaortic soft tissue mass are the most common imaging features of mycotic aneurysms, present in approximately 48 % of cases [54], and may be the only early signs of infection before aneurysm development [58]. Aortic wall calcification and mural thrombus are not common features of infected aneurysms [10, 54]. Other potential imaging findings include peri-aneurysmal gas, vertebral body destruction, psoas abscess formation and renal infarction [47]. Infected aortic aneurysms demonstrate increased tracer uptake on 18 F-FDG PET/CT in both the aneurysm wall and surrounding soft tissue when compared to infection-free aneurysms, which may be a discriminator in equivocal cases [59–61]. Mycotic aneurysms appear on ultrasound as saccular aneurysms with mural thickening [10]. A CT grading system for mycotic aneurysms that correlates with clinical severity and mortality has been proposed by Lai et al. as follows: grade 1 = periaortic inflammation without dilatation; grade 2 = presence of saccular aneurysm; grade 3 = extensive retroperitoneal infection; grade 4 = peri-aneurysmal haemorrhage [62]. Early diagnosis is crucial, as untreated mycotic aneurysms are associated with high mortality from rupture or uncontrolled sepsis [52, 63].

### Tuberculous aortitis

**Fig. 11:**
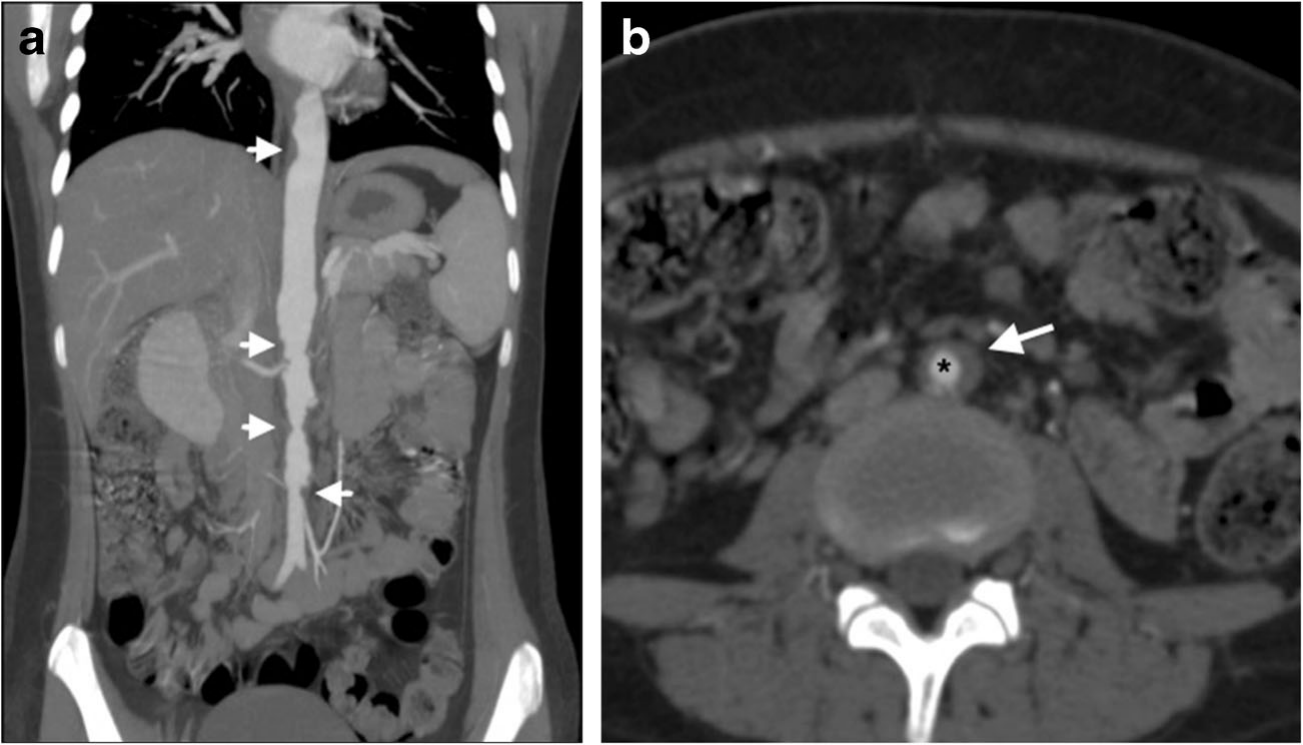
A 15-year-old girl of Indian origin presents with cough, night sweats and back pain. (**a**) A post-contrast CT abdominal angiogram coronal maximum intensity projection (MIP) demonstrates multifocal stenoses of the aortic hiatus and the suprarenal and infrarenal abdominal aorta (*arrowheads*). (**b**) An axial CT abdominal angiogram demonstrates severe luminal narrowing (*) of the infrarenal abdominal aorta, with circumferential periaortic soft tissue (*arrow*). The patient’s sputum cultures grew *M. tuberculosis*, with aortic appearance consistent with tuberculous aortitis

Infection of the aortic wall with *Mycobacterium tuberculosis* is rare. It can be caused by direct extension from adjacent tuberculous infected tissue, or by haematogenous seeding from a remote tuberculous focus, the latter suggesting the presence of disseminated tuberculosis [64]. Tuberculous aortitis commonly affects the distal aortic arch and descending thoracic aorta, presenting with a pseudoaneurysm caused by caseous necrosis of the aortic wall [65]. On cross-sectional imaging, tubercular aortitis often appears as a focal, saccular pseudoaneurysm with multiple lobular outpouchings and an irregular, thickened aortic wall [66]. Perforation of the aorta into adjacent structures such as the oesophagus, pulmonary tree, peritoneal cavity and small bowel have been reported [67]. Aortic dissection and focal aortic wall thickening without aneurysmal dilatation may also occur [66]. In the latter entity, the imaging appearance can be identical to that of chronic periaortitis, appearing as circumferential encapsulation of the aorta with enhancing soft tissue, which may result in symptomatic stenosis (Fig. 11) [68]. Increased tracer uptake in the aortic wall on 18 F-FDG PET/CT has been described in this form of tuberculous aortitis, mimicking chronic periaortitis [57]. The overall prognosis of tuberculous aortitis is poor, with mortality rates as high as 60 %, despite appropriate antitubercular and surgical treatment [67].

### Syphilitic aortitis

**Fig. 12:**
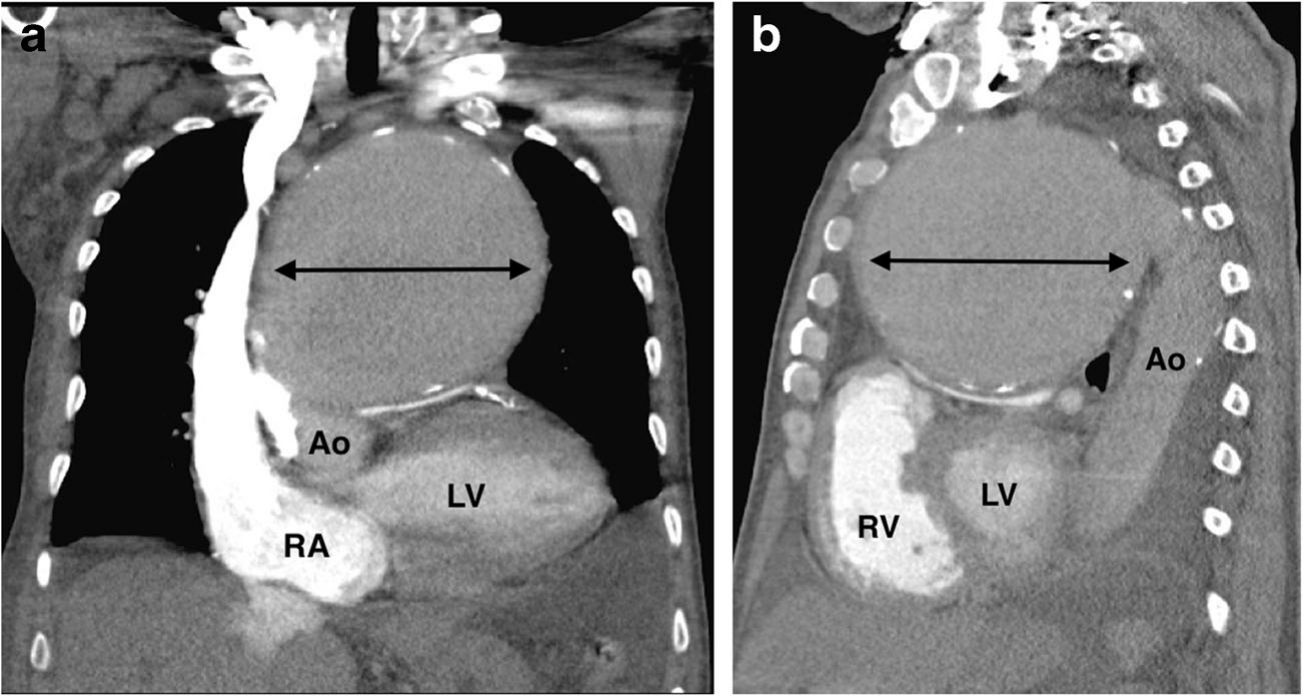
A 70-year-old-man with chest pain and dyspnoea on exertion underwent a CT pulmonary angiogram (CTPA) for suspected pulmonary embolus. (**a**) Coronal and (**b**) sagittal MPRs from the CTPA demonstrate a large, fusiform, peripherally calcified aneurysm (*double arrow*) of the ascending aorta (Ao), causing severe compression of the main pulmonary artery inferiorly and abutting the sternum anteriorly. The patient underwent surgical repair, with pathology-demonstrated classical appearance of syphilitic aortitis, caused by the spirochete *Treponema pallidum. RA = right atrium; RV = right ventricle; LV = left ventricle*

Syphilis is a chronic infection caused by the sexually transmitted spirochete *Treponema pallidum*. Tertiary syphilis is characterized by cardiovascular involvement, along with the presence of gummas and neurological involvement, typically occurring 15 to 30 years after the initial infection. Cardiovascular involvement is caused by endarteritis of the vasa vasorum, and can manifest as aortitis, aortic aneurysm, aortic regurgitation or coronary artery stenosis [47]. The ascending thoracic aorta is the most commonly affected aortic site, followed by the aortic arch [69]. The most common appearance on CT/MR is diffuse aortic mural thickening with associated aneurysm formation, which may be multiple and saccular (Fig. 12) [70]. Syphilitic thoracic aortic aneurysms may grow to considerable size, and can cause sternal or clavicle erosion [71]. The aortic wall may have a double-ring appearance on CT, with hyperdense outer and hypodense inner layers [72]. Chronic inflammation of the aorta leads to intimal fibrosis and calcification, which can give rise to a tree bark appearance of the aortic wall, which may be visible on chest radiographs [47, 73]. Aneurysm rupture, severe aortic insufficiency and coronary artery ostial narrowing are the most common causes of death in patients with syphilitic cardiovascular disease [74]. There are reports suggesting that 18 F-FDG PET/CT may be used to detect aortic involvement at an early stage, before the occurrence of potential life-threatening sequelae of syphilitic aortitis [75, 76].

### Aortic involvement in patients with human immunodeficiency virus (HIV)

HIV causes aortitis though a complex mechanism of infectious and non-infectious processes. It accelerates atherosclerotic disease, which results in both occlusive and aneurysmal aortic disease [77]. There is also a broad spectrum of non-atherosclerotic vasculitides which can occur in patients with HIV, either due to opportunistic infection or directly caused by HIV infection [78]. The imaging features are non-specific, including aortic aneurysms, aneurysms involving multiple large vessels, aortic dissection and occlusive aortic disease [79].

### Choosing an imaging modality

**Fig. 13:**
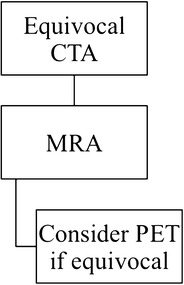
A suggested imaging workflow diagram for suspected infectious aortitis with an equivocal initial CT angiogram [57, 62, 63, 100]

In cases of suspected aortic infection, one must take into account the patient’s clinical condition when selecting an imaging modality. In an emergency situation, a CT aorta angiogram is our initial imaging test of choice. We find that a single post-contrast arterial phase CT acquisition is usually sufficient; an additional post-contrast delayed phase (approximately 70 s) may be useful in cases with suspected haemorrhage or aortic fistula. In the non-emergency setting in our practice, CTA is often the first test performed, with MRA used as a problem-solving tool and for follow-up to monitor treatment. MRA can be used as the initial imaging test, particularly in young patients or those who cannot receive iodinated contrast. We perform 18 F-FDG PET/CT mainly for cases that are equivocal on CT and/or MRA (Fig. 13). Another potential application of PET/CT in this context is in monitoring treatment response by monitoring aortic metabolic activity.

There is a paucity of data on the test properties of the various cross-sectional modalities in the diagnosis of aortic infection; the majority of published case series do not include controls in their analysis. One review of 30 cases of bacterial aortitis with and without aneurysmal dilation reported a sensitivity of 67 % for CTA [63]. A review of the role of 18 F-FDG PET/CT in both infectious and non-infectious aortitis by Bruls et al. reported a sensitivity of 100 % for 18 F-FDG PET/CT in cases of infectious aortitis [57], although the cohort of infected patients in this series was very small.

## Aortic infection post-graft repair

Infection is a rare complication of aortic graft repair, with significant mortality and morbidity. The incidence of prosthetic graft infection post-open aortic repair varies from 0.6 % to 5 %, with mortality rates ranging from 25 % to 88 % [80, 81]. Infection rates are lower with endovascular graft repair that with open surgical approaches, with an incidence of approximately 1 % [82]. The most common microorganism isolated within the first 3 months after an open repair is *Staphylococcus aureus*, with coagulase-negative *Staphylococcus* more common in late infections [83]. A similar flora is implicated in endograft infections, with *Staphylococcus* and *Streptococcus* most commonly isolated (Table 1) [82, 84]. There are several treatment options for infected aortic grafts both endovascular and open, including graft excision with extra-anatomic bypass and in situ reconstruction [85]. Symptoms of aortic graft infection can be non-specific, including recurrent fever and chills, back or chest pain, erythema and swelling. Early diagnosis is crucial, as treatment delays are associated with significant morbidity and mortality [86].

**Fig. 14:**
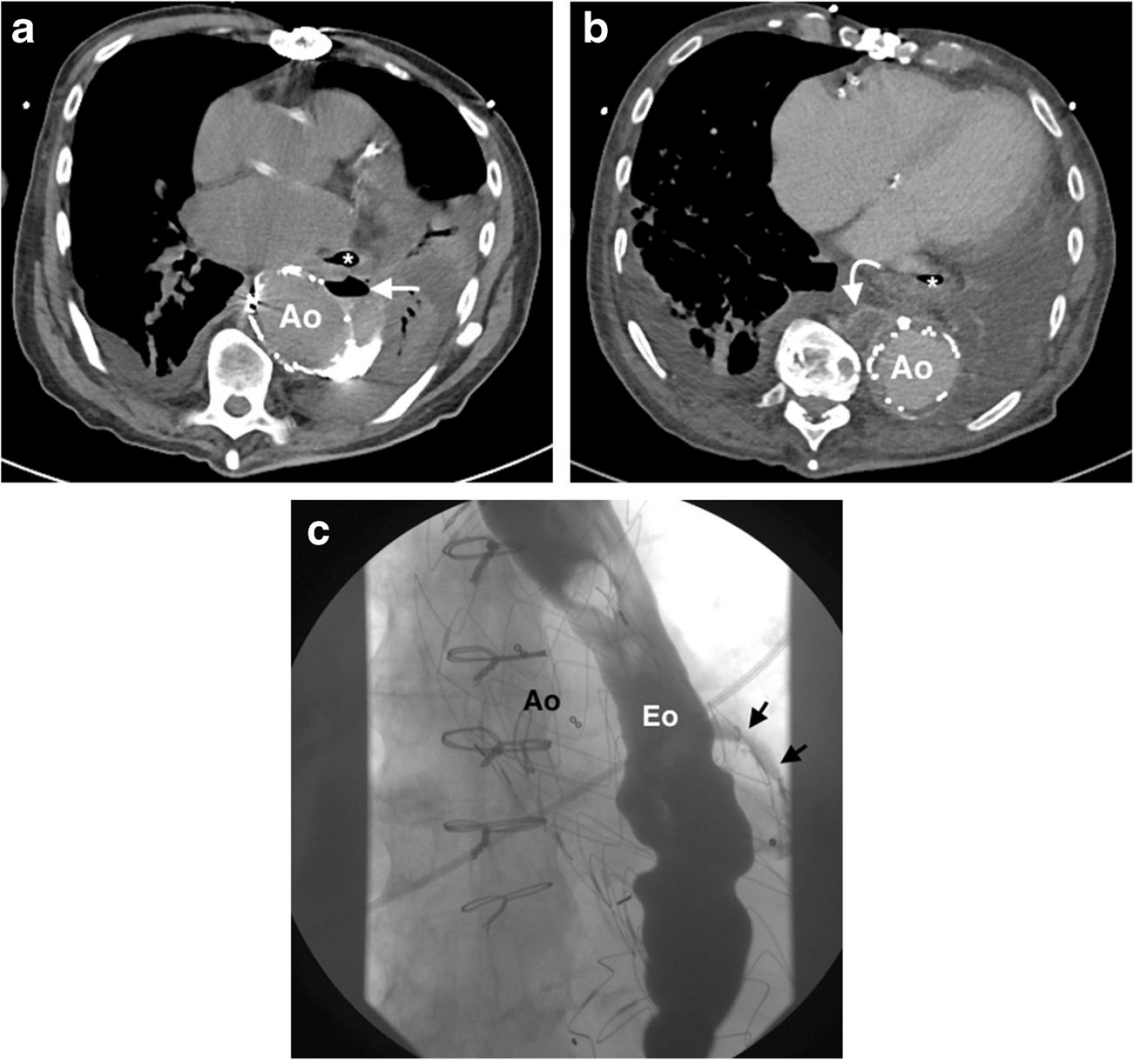
A 65-year-old man presents 6 months post-thoracic endovascular aneurysm repair (TEVAR) with fever and haemoptysis. (**a**) An axial non-contrast CT thorax image demonstrates new air within the excluded aneurysm sac (*arrow*), immediately posterior to the oesophagus (*). There is adjacent left lower lobe consolidation with associated pleural effusion. (**b**) An axial CT of the thorax acquired 70 s post-contrast demonstrates a peripherally enhancing periaortic fluid collection (*curved arrow*) at the inferior aspect of the stent graft consistent with an abscess. (**c**) A barium swallow confirms the presence of an aorto-oesophageal fistula (*arrows*). The overall diagnosis is consistent with a thoracic stent graft infection with an aorto-oesophageal fistula, likely caused by the adjacent left lower lobe pneumonia. The patient died despite surgical intervention, with no definite organism identified. *Ao = descending thoracic aorta; Eo = oesophagus*

**Fig. 15:**
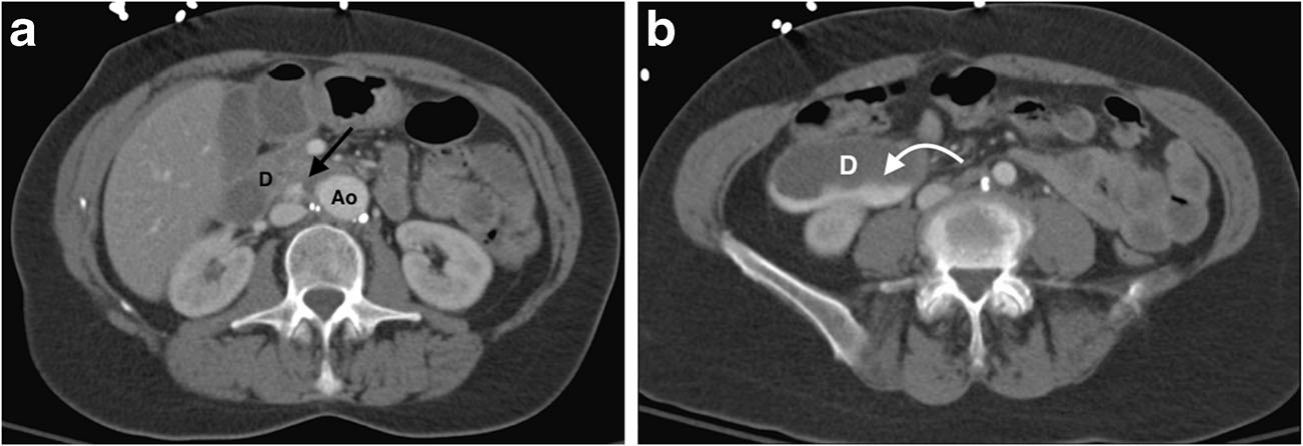
A 62-year-old woman with a history of a previous open abdominal aortic aneurysm graft repair presents with abdominal pain and emesis of brown particulate matter, and was referred for CT angiogram. (**a**, **b**) Axial slices of a CT scan of the abdomen acquired 70 s post-contrast demonstrates a fistula (*arrow*) between the aorta (Ao) and duodenum (d), with layering of high-density material in the duodenum (*curved arrow*). The patient underwent emergency surgery, but unfortunately died. Post mortem culture of the abdominal aorta graft grew gram-negative bacilli, likely *E. coli*

CT is the most commonly used imaging modality for the assessment of aortic graft infection. Signs of infection on CT include persistent or expanding perigraft soft tissue thickening, perigraft fluid collection and gas after the initial postoperative period (Fig. 14) [49, 80, 81]. Persistence of perigraft soft tissue attenuation and fluid beyond 3 months post-open aortic repair is suspicious for graft infection. The presence of perigraft air in the abdominal aorta beyond the initial postoperative period (1 month) is suspicious for, but not pathognomonic of, an aortoenteric fistula [87]. The combination of ectopic gas and focal bowel wall thickening and/or tethering of bowel loops adjacent to the graft site is highly suggestive of an aortoenteric fistula [87]. In rare cases, extravasation of contrast from the aorta into the involved loop of bowel can be demonstrated on CT (Fig. 15). The acquisition of a delayed post-contrast CT (approximately 70 s) can help to demonstrate a fistula, if present. Pseudoaneurysm formation is a serious complication of graft infection, occurring particularly in cases of endovascular graft infection [88, 89].

On of the major challenges is identifying patients with low-grade graft infections; these have a reported sensitivity and specificity on CT of 55–64 % and 86–100 %, respectively [90, 91]. In these cases, CT can provide image guidance for fine needle aspiration to provide a definitive microbiological diagnosis (Table 2).

Tissue characterisation is a useful feature of MRI in cases of suspected graft infection. MRI can distinguish perigraft fluid and inflammation from chronic haematoma based on T1 and T2 signal characteristics and post-contrast enhancement (Table 2). Shahidi et al. compared MRI with indium-111-labelled white blood cell scanning in the diagnosis of aortic graft infection in 59 patients; MRI performed well, with a sensitivity of 68 % and specificity of 97 % [92]. The principal ultrasonographic features of graft infection are the presence of pseudoaneurysms, persistent gas and perigraft fluid collections [80], but there is limited data on its performance in identifying aortic graft infections.

To help overcome some of the shortcomings of CT and MRI in the diagnosis of aortic graft infections, especially low-grade infections, 18 F-FDG PET/CT has been proposed as a useful adjunct, with several series reporting a sensitivity and specificity of 89–100 % and 86–100 %, respectively [93–96]. False positives may occur due to chronic inflammation on the graft surface, appearing as diffuse, low to moderate FDG avidity along the graft, especially in the early postoperative phase (first 3 months) [97, 98]. A focal or heterogeneous pattern of increased FDG uptake along the graft is suggestive of infection [7].

### Choosing an imaging modality

**Fig. 16:**
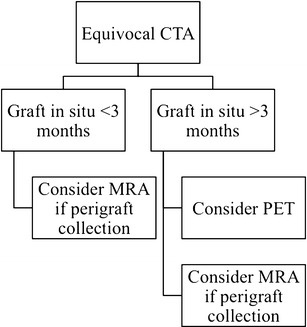
A suggested imaging workflow diagram for suspected prosthetic aortic infection with an equivocal initial CTA [80, 81, 97, 98, 100]

CTA is typically the initial imaging test of choice in cases of suspected aortic graft infection. We find the acquisition of a non-contrast CT phase useful in these cases, as it helps to define the distribution of post-surgical material; for example, felt pledgets commonly used in thoracic aortic repair have high attenuation on CT, and can be confused with a pseudoaneurysm in the absence of a pre-contrast CT [99]. An additional delayed phase (approximately 70 s) post-contrast CT acquisition is beneficial in cases with a suspicion of an aortic fistula, and to evaluate for an endoleak in patients with an endovascular stent graft. The use of MRA in cases of suspected graft infection is often limited by metallic artefacts from the graft material causing local signal loss; these problems are most manifest with endovascular stent grafts. Despite this, MR can play a complementary role to CT in selected cases—for example, using its tissue characterisation ability to help distinguish perigraft fluid, inflammation and haematoma. 18 F-FDG PET/CT is a useful adjunct to CT and MRI, although false positives may occur in the initial post-implantation phase and due to chronic inflammation on the graft surface (Fig. 16) [97, 98].

## Conclusion

The diagnosis of aortic infections requires the integration of clinical, laboratory and imaging features. A multimodal approach to the imaging diagnosis is often required, combining ultrasound, CT, MRI and 18 F-FDG PET/CT, depending on the clinical scenario. The 2015 ESC guidelines on the diagnosis and management of infective endocarditis recommend echocardiography as the initial imaging modality of choice, with the addition of cardiac CT and/or 18 F-FDG PET/CT according to the clinical situation [17]. Current ESC guidelines on the diagnosis and management of aortic diseases recommend the use of either CT or MRI as first-line imaging techniques in cases of suspected aortic infection, with 18 F-FDG PET/CT reserved for special clinical scenarios such as cases of suspected aortic prosthetic infection [100]. It is important that reporting radiologists are vigilant to the various manifestations of aortic infections on cross-sectional imaging, as prompt diagnosis and treatment can be lifesaving.
